# Gradient-based MCMC samplers for dynamic causal modelling

**DOI:** 10.1016/j.neuroimage.2015.07.043

**Published:** 2016-01-15

**Authors:** Biswa Sengupta, Karl J. Friston, Will D. Penny

**Affiliations:** Wellcome Trust Centre for Neuroimaging, Institute of Neurology, University College London, 12 Queen Square, London WC1N 3BG, UK

## Abstract

In this technical note, we derive two MCMC (Markov chain Monte Carlo) samplers for dynamic causal models (DCMs). Specifically, we use (a) Hamiltonian MCMC (HMC-E) where sampling is simulated using Hamilton’s equation of motion and (b) Langevin Monte Carlo algorithm (LMC-R and LMC-E) that simulates the Langevin diffusion of samples using gradients either on a Euclidean (E) or on a Riemannian (R) manifold. While LMC-R requires minimal tuning, the implementation of HMC-E is heavily dependent on its tuning parameters. These parameters are therefore optimised by learning a Gaussian process model of the time-normalised sample correlation matrix. This allows one to formulate an objective function that balances tuning parameter exploration and exploitation, furnishing an intervention-free inference scheme. Using neural mass models (NMMs)—a class of biophysically motivated DCMs—we find that HMC-E is statistically more efficient than LMC-R (with a Riemannian metric); yet both gradient-based samplers are far superior to the random walk Metropolis algorithm, which proves inadequate to steer away from dynamical instability.

## Highlights

• We compare 2 gradient-based MCMC methods for parameter inference in neural mass models.
• These methods are Hamiltonian and Langevin Monte Carlo (HMC-E and LMC-R).
• LMC-R is computationally more efficient while HMC-E proves to be statistically most efficient.
• We propose combining gradient-based and gradient-free samplers for efficient Bayesian inference.

## 1 Introduction

A common problem in neuroimaging is one where we observe some data *y* ≈ *f*(*θ*) with *θ* being a vector of parameters and we are interested in asking how likely the observed data is under a generative model describing *f*(*θ*). The observed data is typically in the form of EEG, MEG or fMRI time series. Dynamic causal models (DCMs) (Friston et al., 2003) provide a portfolio of generative models that can range from detailed biophysical models, like neural mass models (NMMs) (David et al., 2006) to phenomenological models used to explain phase coupling between multiple brain regions (Penny et al., 2009). Bayesian statistics then enable us to compute the posterior density of parameters *π*_*post*_(*θ*|*y*) using Bayes rule, i.e., $\pi_{\mathrm{post}}\left(\theta \left|y\right)\right)=Z^{-1}\left(\pi_{\mathrm{like}}\left(y\left|\theta \right)\right)\pi_{\mathrm{prior}}\left(\theta \right)\right)$ where *π_like_* and *π_prior_* are the likelihood and the priors, respectively, while the partition function Z is a normalisation constant. In what follows, we will drop any dependence on Z, as our interest lies in sampling the distribution, where the requirement that the distribution normalises to unity is not necessary.

When there are many parameters, sampling from the posterior density is computationally intractable. Under such circumstances, one typically approximates the posterior density using a probability density with a fixed form. This converts the problem of evaluating high-dimensional integrals into an optimisation problem (Friston et al., 2003). Variants of this approach are well-known under the remit of variational-Bayesian (VB) inference (Beal, 2003, Wainwright and Jordan, 2008). In this note, we are concerned with a complementary approach where our goal is to estimate the posterior density by obtaining a functional that is easy to sample from (Robert and Casella, 2005)—and is computationally cheap to calculate for the generative models used in DCMs. This is generally in the form of a Metropolis–Hastings sampler that traverses randomly on the parameter space, selecting samples that increase the joint-likelihood of the data under the current parameters (Chumbley et al., 2007). Although conceptually simple, such a random-walk Metropolis algorithm converges slowly to the target posterior density (slow mixing). This is untenable when the likelihood comprises solutions of tens or hundreds of differential equations. More so, for infinite-dimensional dynamical systems governed by partial-differential equations (PDEs).

To alleviate slow mixing (statistical inefficiency), we use the first-order gradient of the joint log-likelihood, sampling from the posterior density using either the Hamiltonian MCMC (HMC-E) method (Duane et al., 1987, Neal, 2010) or the Langevin Monte Carlo algorithm (LMC-E and LMC-R) method (Girolami and Calderhead, 2011). We contrast the performance of both algorithms using a single node NMM, exploiting highly efficient differential equation integrators (CVODES) (Hindmarsh and Serban, 2002) and an adjoint formulation for the gradients (Sengupta et al., 2014), where possible. In brief, our indicative results show that in terms of statistical efficiency, HMC-E followed by LMC-R are strong contenders while random-walk Metropolis–Hastings and LMC-E do not seem to mix at all (non-convergence) for problems with unstable dynamics—the dynamics become stiff and difficult (too slow) for a practical algorithm to integrate.

## 2 Methods

In this section, we briefly describe the generative model (DCM) used to evaluate the sampling schemes and consider generic issues pertaining to numerical integration (solution of differential equations) implicit in evaluating log-likelihoods and their gradients. We then describe the Hamiltonian (HMC-E) scheme—and how potential problems with its tuning can be finessed. Secondly, we consider the Langevin Monte Carlo (LMC-E and LMC-R) scheme which is somewhat simpler. As we will see, LMC and HMC are not separate algorithms—LMC is a limiting case of HMC, i.e., HMC reduces to LMC by taking the integration time to be as small as the step size. What we have pursued in this paper is a comparison of Hamiltonian MC using Euclidean metric and Langevin MC using a Riemannian as well as a Euclidean metric. For the LMC algorithm, our comparisons demonstrate the utility of taking into account the curved nature of the statistical manifold in the design of an efficient sampler. Finally, we use a gradient-free random-walk Metropolis–Hastings algorithm to gauge the efficiency of the gradient-based algorithms. Due to algebraically involved calculations involving third-order tensors, comparison to a Hamiltonian MC using a Riemannian metric will be presented in our forthcoming technical note (Sengupta et al., in preparation).

Custom code was written in MATLAB 2014a (The MathWorks, Inc., USA) to simulate the Markov chains. Unless stated otherwise, out of the 20,000 samples that were collected, the initial 6000 samples were discarded as burn-in (see Appendix A). All computations were performed on an Intel Xeon W3570 workstation with 12 GB RAM. Due to different computer architectures and number of samples collected, the simulations in this paper are not comparable to those reported in Sengupta et al. (2015). The source code will be released as a general purpose ‘Monte Carlo inference’ toolbox for SPM (Statistical Parametric Mapping; http://www.fil.ion.ucl.ac.uk/spm/).

### 2.1 Neural mass models

In order to test the inference schemes, we use a single node neural mass model (NMM) based on the DCM proposed by David et al. (2006) to create a synthetic dataset. These DCMs are generally more nonlinear than DCMs used for generative modelling of fMRI time series.

The NMM comprises nine ordinary differential equations (ODEs) of hidden neuronal states *x*(*t*) that are a first-order approximation to delay-differential equations (DDEs), i.e., using *x*(*t* − *δ*) = *x*(*t*) − *δẋ*(*t*) (Eq. (1)). There are ten parameters ({*δ*, *g*, *h*, *τ*, *u*} ⊆ *θ* with *δ* (intrinsic delay), {*g*_1 … 4_} (connection strengths), *h*_*e*/*i*_ (maximum amplitude of post-synaptic potential), *τ*_*e*/*i*_ (rate-constant of the membrane) and *u* (input to the neural population) that govern the flow in three neural populations, namely, inhibitory interneurons (*x*_7_), spiny-stellate (*x*_1_) and pyramidal neurons (*x*_9_). In deterministic DCM, these hidden states are not unknown quantities but respond deterministically to exogenous input according to the differential equations with unknown parameters. By integrating the differential equations and assuming additive Normal noise, the likelihood of observing any data can be modelled as a multi-variate Normal density (Eq. (2)).

(1) $$\begin{array}{l} {\dot{x}}_{1}\left(t\right)=x_{4}\left(t\right)\\ {\dot{x}}_{2}\left(t\right)=x_{5}\left(t\right)\\ {\dot{x}}_{3}\left(t\right)=x_{6}\left(t\right)\\ {\dot{x}}_{4}\left(t\right)=\frac{h_{e}\left(g_{1}\left(\frac{1}{e^{-0.56x_{9}\left(t-\delta \right)}+1}-0.5\right)+u\right)}{\tau_{e}}-\frac{x_{1}\left(t\right)}{{\tau_{e}}^{2}}-\frac{2x_{4}\left(t\right)}{\tau_{e}}\\ {\dot{x}}_{5}\left(t\right)=\frac{g_{2}h_{e}\left(\frac{1}{e^{-0.56x_{1}\left(t-\delta \right)}+1}-0.5\right)}{\tau_{e}}-\frac{x_{2}\left(t\right)}{{\tau_{e}}^{2}}-\frac{2x_{5}\left(t\right)}{\tau_{e}}\\ {\dot{x}}_{6}\left(t\right)=\frac{g_{4}h_{i}\left(\frac{1}{e^{-0.56x_{7}\left(t-\delta \right)}+1}-0.5\right)}{\tau_{i}}-\frac{x_{3}\left(t\right)}{{\tau_{i}}^{2}}-\frac{2x_{6}\left(t\right)}{\tau_{i}}\\ {\dot{x}}_{7}\left(t\right)=x_{8}\left(t\right)\\ {\dot{x}}_{8}\left(t\right)=\frac{g_{3}h_{e}\left(\frac{1}{e^{-0.56x_{9}\left(t-\delta \right)}+1}-0.5\right)}{\tau_{e}}-\frac{x_{7}\left(t\right)}{{\tau_{e}}^{2}}-\frac{2x_{8}\left(t\right)}{\tau_{e}}\\ {\dot{x}}_{9}\left(t\right)=x_{5}\left(t\right)-x_{6}\left(t\right) \end{array}$$

Priors on all parameters conform to a Gamma distribution (Table 1), where (by construction) approximately 46%–50% of parameters sampled result in unstable dynamics, marked by positive real eigenvalues of the Jacobian matrix. This ensured that the inference algorithm can steer away from dynamical instability. Although pairwise co-dimension-2 bifurcation analysis was performed (using numerical continuation), co-dimension-10 bifurcations are difficult to chart. Thus, the shape and scale of the Gamma prior were determined numerically by integrating 200,000 NMMs and evaluating the eigenvalues of the Jacobian at the nearest fixed points. The eigenvalues were then used to adjust the prior distribution, such that the sampled parameters produce unstable dynamics. The fixed-point equations were solved using a Trust-Region Dogleg method (Nocedal and Wright, 2006). The initial values are sampled from the prior and are guaranteed to generate parameter sets that emit dynamically stable models.

Contrary to David et al. (2006), where the input was modelled as a combination of a Gamma density function and a discrete cosine set, we used a simpler Heaviside step function to perturb the spiny-stellate cells. This was done to mimic the inputs used during bifurcation analysis. Differential equations were integrated using CVODES (Hindmarsh and Serban, 2002) using implicit backward-differentiation formulas (BDFs). The resulting nonlinear equations were solved using Newton’s method. Initial simulations established that direct solvers based on dense matrices were computationally more efficient than the three pre-conditioned Krylov (iterative) solvers (GMRES, Bi-CGStab and TFQMR) (Golub and Van Loan, 2012). We anticipate that for larger dynamical systems (e.g., a 10-node NMM), iterative solvers would be more efficient. The absolute and relative tolerances of the integrators were both fixed at 10^− 3^.

### 2.2 Sensitivity analysis and adjoints

The efficiency of gradient-based MCMC methods rests on the evaluation of the gradient of the joint log-likelihood function. There are two methods for computing this gradient: (a) forward sensitivity analysis or (b) adjoint sensitivity analysis as described in Sengupta et al. (2014). Given that the forward dynamics can be unstable by design, all inference results considered in this note are based on forward sensitivities. DCMs in general are stable, therefore we show speedup results for stable DCMs when using adjoints for computing gradients. The joint log-likelihood function J reads,

(2) $$\begin{array}{c} J=-\frac{1}{2}\ln\left(\left|\Sigma \right|\right)-\frac{T}{2}\ln\left(2\pi \right)-\frac{1}{2}{\left(x_{9}\left(\theta \right)-y\right)}^{T}\Sigma^{-1}\left(x_{9}\left(\theta \right)-y\right)\\ +\ln\left(\frac{1}{\Gamma \left(k_{1}\right){k_{2}}^{k_{1}}}\theta^{k_{1}-1}e^{-\frac{\theta }{k_{2}}}\right) \end{array}$$

where, Σ is the observation noise co-variance matrix with diag(Σ) = 0.0625, *T* is the total datapoints observed, *x*_9_(*θ*) is the predicted pyramidal cell voltage, *y* is the observed pyramidal cell voltage and *θ* is a vector of parameters. The fourth term represents the log-Gamma priors on the parameters where *k*_1_ and *k*_2_ are the shape and scale of the Gamma density, respectively. The gradient then reads,

(3) $$\frac{dJ}{d\theta }=-{\left(x_{9}\left(\theta \right)-y\right)}^{T}\Sigma^{-1}\frac{dx_{9}}{d\theta }+\frac{k_{1}-1}{\theta }-\frac{1}{k_{2}}$$

The forward-sensitivity method involves using Gronwall’s theorem (Sengupta et al., 2014) to compute the state sensitivities, as a function of time. This method is less efficient than using the adjoint of the dynamical system: in brief, a single solution of the nonlinear (NMM) ODE and a single (linear) adjoint ODE provides the gradient, where:

(4) $$\frac{dJ}{d\theta }=\int_{0}^{T}\left(\frac{\partial j}{\partial \theta }-\lambda^{T}\frac{\partial f}{\partial \theta }\right)dt$$

*λ*^*T*^ is the adjoint-vector, *j*(*θ*) is the right hand side of Eq. (2) and *f*(*θ*) represents the differential equations that form the single-node NMM. There are two remarks that can be made about the adjoint formulation. First, regardless of whether the underlying differential equation is linear or nonlinear, the adjoint method requires the integration of a single linear equation—the computational efficiency is inherent in the structure of the equation. Second, the appearance of a transpose on the adjoint vector implies that the flow of information in the system of equations is reversed; it is in this sense that the adjoint equations are integrated backwards in time. The proof for Eq. (4) is available in Sengupta et al. (2014).

Sensitivity analysis was performed using CVODES and cross-checked manually using the code-base in Sengupta et al. (2014). Both methods yielded identical results. Forward mode automatic differentiation using ADiMat (Bischof et al., 2002) yielded sub-optimal execution time in comparison to numerical differentiation for computing intermediate sensitivity operators. We anticipate that the computational efficiency of automatic differentiation would be prominent for larger dynamical systems.

### 2.3 Algorithm A—Hamiltonian Monte-Carlo

The random walk Metropolis–Hastings algorithm has slow mixing because of the inherent random walks (Chumbley et al., 2007). Hybrid or Hamiltonian Monte Carlo (HMC-E) resolves this issue by equipping the proposal distribution with a dynamics that reduces the amount of random-walk—correcting for any numerical error in the integration of this dynamics using a Metropolis–Hastings acceptance criteria (Duane et al., 1987, Neal, 2010). Hamiltonian dynamics is a reformulation of Newton’s second law of motion; where the evolution of any energy-conserving system can be described with a pair of first order ODEs. In the present context, the Hamiltonian is a function of the unknown parameters, where its potential energy is given by the negative log-likelihood.

Heuristically, we want to find a way of exploring parameter space to evaluate the log-likelihood; rejecting or accepting samples using Metropolis–Hastings criterion to approximate the posterior distribution. The slow mixing of a random walk Metropolis–Hastings can be alleviated if we use the local gradients of the log-likelihood to explore the parameter space in a more informed fashion. Hamiltonian Monte Carlo techniques do this by using the trajectory implied by Hamiltonian dynamics when the potential energy is the log-likelihood (cf., the trajectory of a frictionless marble rolling over the log-likelihood landscape). However, there is a problem: we do not know the form or roughness of this landscape and the momentum of the marble must be tuned. This induces tuning parameters, which themselves have to be optimised—as we will see below.

In detail, the total energy of the Hamiltonian H, with parameters *θ* and their respective momentum *ρ*, reads

(5) $$H\left(\theta ,\rho \right)=E_{\mathrm{pot}}\left(\theta \right)+E_{\mathrm{kin}}\left(\theta ,\rho \right)$$

where, E_*pot*_ is the potential energy and E_*kin*_ is the kinetic energy. Then, by Hamilton’s principle, we have

(6) $$\begin{array}{l} \dot{\theta }=\frac{\partial H}{\partial \rho }\\ \dot{\rho }=-\frac{\partial H}{\partial \theta } \end{array}$$

In order to use Hamilton’s equations in an MCMC setting, the dynamics need to be reversible so as to satisfy detailed balance, and their solutions should be volume preserving. Numerical integrators must be used solely because analytic results for such a problem are not available. Fortunately, as we describe below, symplectic integrators offer highly accurate numerical approximations that are both reversible and exactly preserve volume, which means they can also be made statistically exact with the application of a Metropolis correction.

Reversibility is guaranteed by Picard’s theorem, which says that for first-order differential equations, the Hamiltonian flow is bijective, wherein invertibility of the flow-map guarantees the reversibility of the dynamics. Secondly, Hamiltonian systems are symplectic, i.e., the volume enclosed by nearby solutions is constant. This is a consequence of the Liouville’s theorem, which says that the vector field prescribed by Hamilton’s equation has zero-divergence (Leimkuhler and Reich, 2004). With these constraints in mind, we use a symplectic reversible integrator known as the ‘leapfrog scheme’ (Störmer-Verlet) to simulate the Hamiltonian of our statistical model. We simulate an iteration of this scheme with step-size *ε*, moving from (*θ*_0_, *ρ*_0_) to (*θ*_*n*_, *ρ*_*n*_) via a two-step process,

(7) $$\begin{array}{c} \rho^{1/2}=\rho_{0}-\frac{\varepsilon }{2}\nabla_{\theta }H\left(\theta_{0},\rho^{1/2}\right)\\ \overset{⌢}{\theta }=\theta_{0}+\frac{\varepsilon }{2}\nabla_{\rho }H\left(\theta_{0},\rho^{1/2}\right)+\frac{\varepsilon }{2}\nabla_{\rho }H\left(\overset{⌢}{\theta },\rho^{1/2}\right)\\ \rho_{n}=\rho^{1/2}-\frac{\varepsilon }{2}\nabla_{\theta }H\left(\overset{⌢}{\theta },\rho^{1/2}\right) \end{array}$$

For a Hamiltonian with a mass-matrix *M* and momentum $\pi \left(\rho \right)\sim N\left(0,M\right)$, the *D* dimensional joint density over parameters and their momentum can be factored as *π*(*θ*, *ρ*) = *π*(*θ*)*π*(*ρ*), such that the Hamiltonian becomes separable,

(8) $$\begin{array}{c} H\left(\theta ,\rho \right)=-J\left(\theta \right)+\frac{1}{2}\rho^{T}M^{-1}\rho +\frac{1}{2}\log\left({\left(2\pi \right)}^{D}\left|M\right|\right)\\ :\pi \left(\theta \right)\propto \int \exp\left(-H\left(\theta ,\rho \right)\right)d\rho \end{array}$$

Eq. (6) now simply reads $\dot{\theta }=M^{-1}\rho$ and $\dot{\rho }=\nabla_{\theta }J\left(\theta \right)$. We set *M* to be a positive-definite diagonal matrix with the leapfrog updates in Eq. (7) now defined as

(9) $$\begin{array}{c} \rho \left(t+\frac{\varepsilon }{2}\right)=\rho \left(t\right)+\frac{\varepsilon }{2}\nabla_{\theta }J\left(\theta \left(t\right)\right)\\ \theta \left(t+\varepsilon \right)=\theta \left(t\right)+\varepsilon M^{-1}\rho \left(t+\frac{\varepsilon }{2}\right)\\ \rho \left(t+\varepsilon \right)=\rho \left(t+\frac{\varepsilon }{2}\right)+\frac{\varepsilon }{2}\nabla_{\theta }J\left(\theta \left(t+\varepsilon \right)\right) \end{array}$$

To correct the errors introduced by numerical integration, we subject the samples to the Metropolis acceptance criteria *s* < (1 ∧ exp(H_*old*_ − H_*new*_)) where $s\sim U\left(0,1\right)$.

Frequently, one wants to infer parameters that satisfy certain constraints. While constraints such as positivity are easily enforced using log-transforms, there are times where one has a priori knowledge of the parameter space that is enforceable either via truncated priors or vector functions representing the constraint function. In HMC, such constraints could be applied using an infinite potential but this fails because the gradient of the potential is undefined at the constraint surface (Betancourt and Stein, 2011). As shown by Betancourt and Stein (2011), we appeal to a classical result in mechanics which says that the components of the momentum vector that are perpendicular to the constraint surface reflect, while preserving the value of the Hamiltonian. This is known as the *specular reflection* of the momentum. As soon as the constraint is violated, we compute the normal $\hat{n}$ and replace the momentum update with a reflection of the momentum. For a smooth constraint *C*(*θ*), this amounts to,

(10) $$\begin{array}{c} \hat{n}=\frac{\nabla C\left(\theta \right)}{\left|\nabla C\left(\theta \right)\right|}\\ \rho_{\mathrm{new}}=\rho_{\mathrm{old}}-2\left(\rho_{\mathrm{old}}\cdot \hat{n}\right)\hat{n} \end{array}$$

### 2.4 Tuning of hyperparameters in HMC

There are two hyper-parameters ({*ε*, *L*} ⊆ *ζ*) for the HMC algorithm—step-size (*ε*) and the number of leapfrog steps (*L*). In the initialisation phase for the HMC, we fix *L* and bisect *ε* to achieve a target acceptance rate of 0.65 (Beskos et al., 2013). We then begin sampling and choose *ε* and *L* every *l*-th sampling step to maximise the expected squared jumping distance (ESJD) (Pasarica and Gelman, 2010) as,

(11) $$g\left(\zeta \right)=\frac{E_{\zeta }{\left\|\theta^{t+1}-\theta^{t}\right\|}^{2}}{\sqrt{L}}$$

Maximising such a functional not only guarantees minimising the first-order correlation of the drawn sample but also bounds the computational time (Wang et al., 2013). We use a Gaussian process (Rasmussen and Williams, 2005) to approximate the unknown function *g*(⋅) by observing it in discrete sampling events (*l* = 10) such that

(12) $$\begin{array}{c} g\left(\cdot \right)\sim GP\left(0,k\left(\cdot ,\cdot \right)\right)∋g\left(\cdot \right)\left|\zeta \right)\sim N\left(\mu_{i}\left(\zeta \right),\sigma_{i}^{2}\left(\zeta \right)\right)\\ \mu_{i}\left(\zeta \right)=k^{T}{\left(K+\sigma_{\text{mod}}^{2}I\right)}^{-1}g_{i}\\ \sigma_{i}^{2}\left(\zeta \right)=k\left(\zeta ,\zeta \right)-k^{T}{\left(K+\sigma_{\text{mod}}^{2}I\right)}^{-1}k \end{array}$$

where *k* = [*k*(*ζ*, *ζ*_1_) ⋯ *k*(*ζ*, *ζ*_*i*_)]^*T*^, *σ*_mod_^2^ is the GP observation variance and

(13) $$K=\left[\begin{array}{ccc} k\left(\zeta_{1},\zeta_{1}\right) & \ldots & k\left(\zeta_{1},\zeta_{i}\right)\\ \vdots & \ddots & \vdots \\ k\left(\zeta_{i},\zeta_{1}\right) & \cdots & k\left(\zeta_{i},\zeta_{i}\right) \end{array}\right]$$

The covariance matrix *k*(⋅,⋅) implements automatic relevance determination (ARD) with $k\left(\zeta_{i},\zeta_{j}\right)=e^{-\frac{1}{2}\zeta_{i}^{T}\Sigma_{GP}^{-1}\zeta_{j}}$. The characteristic length-scales for Σ_*GP*_ = *diag*([*λ*(*ζ*_max_ − *ζ*_min_)]^2^). After approximating the ESJD using a GP, we proceed by using the posterior mean and co-variance kernel to formulate and maximise an acquisition function that enables us to select the best possible *ζ* for the next sampling interval. The upper confidence bound (*ψ*) is one such acquisition function that trades-off exploration and exploitation in the *ζ* space (Srinivas et al., 2010, Srinivas et al., 2012),

(14) $$\begin{array}{c} \psi \left(\zeta ,v\right)=\mu_{i}\left(\zeta ,v\right)+a_{i}b_{i+1}^{1/2}\sigma_{i}\left(\zeta \right)\\ a_{i}=\frac{1}{\sqrt{\max\left(\frac{i-\phi +1}{\nu +1},1\right)}}\\ b_{i+1}^{1/2}=2\log\left(\frac{{\left(i+1\right)}^{\varsigma /2+2}\pi^{2}}{3\kappa }\right)\\ \zeta_{i+1}=\underset{\zeta }{\arg\;\max}\psi \end{array}$$

where *v* is a scalar-invariance parameter that is estimated automatically and *a* enforces diminishing adaptation as that in adaptive MCMC (Roberts and Tweedie, 1996). Parameters specific to this algorithm are given in Table 2.

This concludes our description of the Hamiltonian scheme.

### 2.5 Algorithm B—Langevin Monte Carlo algorithm

The Hamiltonian scheme above uses local gradients to inform the exploration of the log-likelihood landscape; however, the trajectories ignore the curved statistical manifold—local curvature and anisotropy of the landscape. This can be overcome by using a Langevin Monte Carlo (LMC-R) scheme that, effectively, models both flow and diffusion over the log-likelihood landscape. Theoretically, the LMC scheme is a limiting case of HMC; if we consider a single leapfrog step (Eq. (9)), the update equations for HMC reduces to

(15) $$\theta \left(t+\varepsilon \right)=\theta \left(t\right)+\frac{\varepsilon^{2}}{2}M^{-1}\nabla_{\theta }J\left(\theta \left(t\right)\right)+\varepsilon M^{-1}\rho \left(t\right)$$

Therefore, it is easy to see that in such a scenario, HMC reduces to a pre-conditioned Langevin diffusion (Eq. (15)). The convergence of the HMC is determined by the structure of the mass matrix, *M* that is set to an identity matrix, i.e., HMC algorithm assumes that each parameter changes isotropically in the parameter space. When *M* is set to an identity matrix and Hamiltonian dynamics is absent, a related update scheme—LMC with an Euclidean metric (LMC-E)—follows,

(16) $$\theta \left(t+\varepsilon \right)=\theta \left(t\right)+\frac{\varepsilon^{2}}{2}\nabla_{\theta }J\left(\theta \left(t\right)\right)+\varepsilon z\left(t\right)$$

where, *z*(*t*) represents a standard normal variate. In the LMC-E, the drift defines the direction of the proposal based on the Euclidean form of the gradient along with using an isotropic form for the diffusion. Often, a pre-conditioning matrix for the gradient is introduced (akin to numerical analysis where pre-conditioning reduces condition number) to account for correlation among parameters. But how to select this matrix in a rigorous and principled manner remains unclear.

One way forward is to use adaptive MCMC (Haario et al., 2001) methods to prune the mass matrix (pre-conditioner), while the one that we adopt here is an information geometric trick (Girolami and Calderhead, 2011). A convenient way to alleviate parametric scaling problems is to use the natural gradient of the log-likelihood, which turns out to be the Fisher information matrix. In doing so, we not only have a principled recipe for deriving the mass matrix but also reduce the computational complexity of running a symplectic numerical integrator required for the HMC algorithm.

Consider the Langevin diffusion equation on a manifold,

(17) $$d\theta \left(t\right)=\frac{1}{2}{\tilde{\nabla }}_{\theta }J\left(\tilde{\theta }\left(t\right)\right)dt+d\tilde{b}\left(t\right)$$

where, ${\tilde{\nabla }}_{\theta }J\left(\theta \left(t\right)\right)$ is the natural gradient (Amari and Douglas, 1998). It is known that the stochastic dynamics of Brownian motion on a manifold is governed by the Laplace–Beltrami operator (Hsu, 2002). The formal proof is provided in Appendix B. Expanding Eq. (17) via differentiation and first-order discretisation yields,

(18) $$\begin{array}{c} \mu \left(\theta_{k},\varepsilon \right)=\theta_{k}+\frac{\varepsilon^{2}}{2}{\tilde{\nabla }}_{\theta }J{\left(\theta \right)}_{k}+\varepsilon^{2}\Lambda \left(\theta_{k}\right)\\ \lambda_{i}\left(\theta_{k}\right)=\frac{1}{2}\sum_{j}\frac{\partial }{\partial \theta_{j}}{\left\{G^{-1}\left(\theta_{k}\right)\right\}}_{ij} \end{array}$$

where we have used the fact that ${\tilde{\nabla }}_{\theta }J\left(\theta \right)=G{\left(\theta \right)}^{-1}\nabla_{\theta }J\left(\theta \right)$. *G* is the metric tensor. With L as the log-likelihood and assuming a constant metric for computational efficiency (although losing accuracy) we have,

(19) $$\begin{array}{c} \theta \left(t+\varepsilon \right)=\theta \left(t\right)+\frac{\varepsilon^{2}}{2}G^{-1}\left(\theta \left(t\right)\right)\nabla_{\theta }L\left(\theta \left(t\right)\right)+\varepsilon \sqrt{G^{-1}\left(\theta \left(t\right)\right)}z\left(t\right)\\ G\left(\theta \right)={\frac{dx}{d\theta }}^{T}\Sigma^{-1}\frac{dx}{d\theta }-\frac{d^{2}\pi_{\mathrm{prior}}\left(\theta \right)}{d\theta^{2}}\\ ={\frac{dx}{d\theta }}^{T}\Sigma^{-1}\frac{dx}{d\theta }-\frac{1-k_{1}}{\theta^{2}} \end{array}$$

This is the Langevin Monte Carlo algorithm (LMC-R) with *z*(*t*) representing a standard normal variate. In our simulations, we have set *ε* = 0.75.

This concludes our description of the Langevin scheme.

### 2.6 Algorithm C—random walk Metropolis–Hastings algorithm

The random walk Metropolis (MH) is the most common MCMC algorithm for Bayesian inference. Given a current value *θ_i_* of a *d*-dimensional Markov chain, the next value is chosen according to a proposal distribution $\tilde{\theta }\sim \pi \left(\tilde{\theta }\left|\theta_{i}\right)\right)$. We choose this to be a standard multi-variate Normal. The sample is then accepted with probability,

(20) $$\alpha =1\wedge \frac{\pi \left(y\left|\tilde{\theta }\right)\right)\pi \left(\tilde{\theta }\right)\times \pi \left(\theta \left|\tilde{\theta }\right)\right)}{\pi \left(y\left|\theta \right)\right)\pi \left(\theta \right)\times \pi \left(\tilde{\theta }\left|\theta \right)\right)}$$

∧ denotes minimum between the left and the right arguments. If *s* ≼ *α* where $s\sim U\left(0,1\right)$ we set $\theta_{i+1}=\tilde{\theta }$. Otherwise, we set *θ*_*i* + 1_ = *θ*_*i*_. The above formula embodies the notion that any proposal that takes the chain closer to a local mode is always accepted, while any other proposal is accepted with the probability equal to the relative densities of the posterior at the proposed and the current values. The covariance of the standard multi-variate Normal distribution is set to an identity matrix pre-multiplied by 0.57 (2.4^2^/10).

This concludes our description of the MCMC samplers.

### 2.7 Convergence criterion

In order to gauge whether the Markov chains have converged to the invariant distribution, we use spectral analysis of Geweke (1992). For this one takes the first 10% of the chain post burn-in (*C_A_*) and the last 50% of the chain (*C_B_*) to construct two estimators for mean parameter value and their respective asymptotic variances (*σ*_*A*_ and *σ*_*B*_) using the spectral density *S*_*h*_(0),

(21) $$\begin{array}{c} \ell_{A}=\frac{1}{C_{A}}\sum_{t=1}^{C_{A}}h\left(\theta^{\left(t\right)}\right),\ell_{B}=\frac{1}{C_{B}}\sum_{t=T-C_{B}+1}^{T}h\left(\theta^{\left(t\right)}\right)\\ S_{h}\left(w\right)=\frac{1}{2\pi }\sum_{t=-\infty }^{t=+\infty }\mathrm{cov}\left(h\left(\theta^{\left(0\right)}\right),h\left(\theta^{\left(t\right)}\right)\right)\exp\left(itw\right) \end{array}$$

*T* is the total number of samples that were collected. One can then construct test statistics (*t*-test; when *c*_*A*_, *c*_*B*_ → ∞ the *t*-test can be approximated using the standard normal *Z* score) to assess the quality of the initial and the final parts of the Markov chain as,

(22) $$\frac{\sqrt{T}\left(\ell_{A}-\ell_{B}\right)}{\sqrt{\frac{\sigma_{A}^{2}}{c_{A}}+\frac{\sigma_{B}^{2}}{c_{B}}}}:C_{A}=c_{A}T,C_{B}=c_{B}T\;\mathrm{and}\;c_{A}+c_{B}<1$$

If the samples are drawn from a stationary part of the chain, then the two means are equal and the *Z*-score has an asymptotically standard Normal distribution. To visually aid the demonstration of this convergence criterion, Fig. 5 shows what happens to the *Z*-scores when successively larger numbers of iterations are discarded from the beginning of the chain obtained from the HMC-E and LMC-R samplers. Each parameter chain for the individual sampler was divided into 80 bins and Geweke’s *Z*-score was repeatedly calculated. The first *Z*-score is calculated with all of the iterations in the chain while the second is calculated after discarding the first segment, the third after discarding the first two segments and continuing until half of the posterior samples collected have been discarded. The plot never discards more than half the chain. Excursions outside the boundaries of ± 2 are indicative of non-convergence. Due to the inability of MH and LMC-E to steer away from dynamic instability, these samplers were not subjected to convergence analysis.

This completes our description of the numerics, the MCMC schemes we wanted to evaluate in this work along with the description of a univariate convergence indicator. We will now look at their relative performance using simulated data, where we know the true values of the parameters.

## 3 Results

**Fig. 1:**
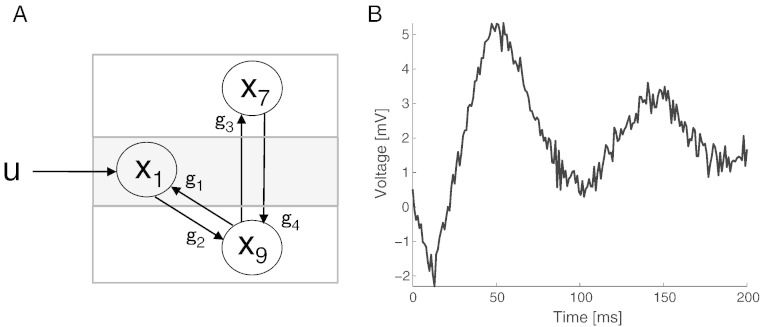
A single node neural mass model (NMM). (A) The forward model consists of 3 neural populations—pyramidal (*x_9_), inhibitory interneuron (x_7_) and spiny-stellate cells (x_1_) connected by linearised delay links (g*_1_, *g*_2_, *g*_3_ and *g*_4_) with *u* serving as a Heaviside input. (B) The pyramidal cell voltage comprises the only observable model. This trace was generated using the parameters in Table 1.

We used a single node NMM (Fig. 1A) to compare the computational efficiency of two gradient-based MCMC samplers, Hamiltonian MCMC (HMC-E) and Langevin Monte Carlo algorithm (LMC-E and LMC-R). To do this, we created synthetic data where we perturbed the NMM using a Heaviside step function, eliciting a stable pyramidal cell voltage (Fig. 1B). Using the pyramidal cell voltage (plus observation noise) as the measured response, the task of the MCMC samplers was to infer the posterior density of parameters. The observation model assumed a Normal likelihood with parameters sampled from a Gamma (prior) distribution (see Methods; Table 1).

**Fig. 2:**
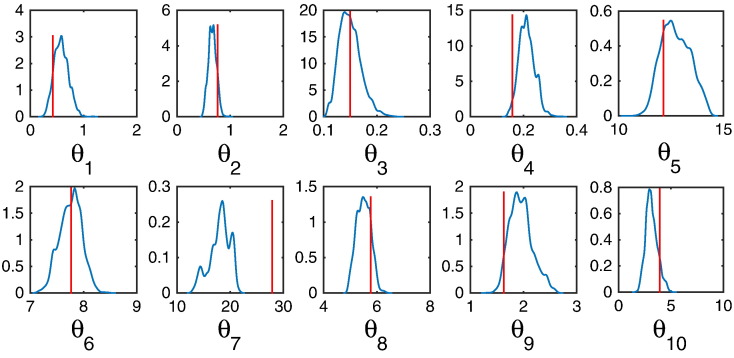
Parameter inference using HMC-E. Kernel density estimates of parameter posteriors; 6000 iterations were excluded as burn-ins, out of 20,000 samples. The red line indicates the true parameter.

Using 30% of the total samples drawn as burn-ins, the HMC algorithm introduces an auxiliary variable (momentum), wherein Hamilton’s equation of motion are integrated to simulate the dynamics of the parameters (position) on the posterior landscape. The end result of integrating the Hamiltonian dynamics yields the posterior density of the parameters. Based on 14,000 samples, the HMC algorithm successfully traverses the parameter space to yield the posterior distributions shown in Fig. 2. All but one parameter (no. 7) is not under the support of the posterior density. This can be alleviated simply by collecting more samples.

The efficiency of a MCMC sampler is defined as the ratio of the computation time and the number of effective samples produced in that time. The effective sample size (ESS) for each parameter is calculated using $ESS=R{\left\{1+2\sum_{q}\gamma \left(q\right)\right\}}^{-1}$, where *R* is the number of posterior samples post-burn-in and $\sum_{q}\gamma \left(q\right)$ is the sum of *Q* monotonic auto-correlations. This auto-correlation is estimated using the initial monotone sequence estimator (Theorem 3.1 in Geyer, 1992). The minimum ESS reflects the number of samples that are effectively uncorrelated across all parameters. Similarly, the time normalised (wall-time/minimum ESS) ESS tells us how much time we effectively spend sampling a single uncorrelated sample, providing us with worst-case performance measure (Cormen et al., 2001). As noted in Table 3, the HMC algorithm produces on average (over 10 parameters) 95 uncorrelated samples while the nESS is around 496 min per sample. The primary reason for this expenditure is the selection of small step sizes (up to 0.0001) and large leap-frog steps (up to 150) for each MCMC iteration. Almost certainly, the computational cost could have been reduced by decreasing the leap-frog steps and/or increasing the step-size. This was deliberately avoided to quantify the worst-case behaviour of this algorithm. Towards the end of the MCMC sampling, although the leap-frog step collapses to a minimum, the GP-UCB optimiser still favours the smallest time-steps.

**Fig. 3:**
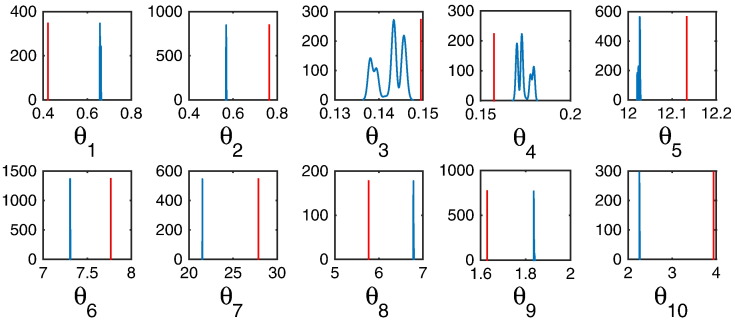
Inference using LMC-E. (A) Kernel density estimates of parameter posteriors; 6000 iterations were excluded as burn-ins, out of 20,000 samples. The red line indicates the true parameter.

Using the Euclidean form of the LMC (LMC-E) shows poor or rather no mixing of the Markov chain (Fig. 3), compared to HMC-E (Fig. 2)—the convergence of LMC-E is suspect. This is demonstrated by the posterior density attaining a Dirac-delta type distribution, with many true parameters not being under the support of the posterior distribution. The average number of un-correlated samples decreases substantially to 4 samples (Table 3). This straightforward comparison demonstrates the necessity of introducing constraints (using Hamilton’s equation) on the motion of the otherwise randomly diffusing parameter.

**Fig. 4:**
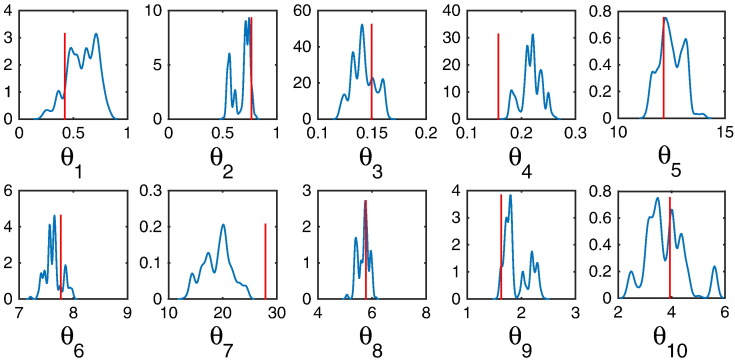
Inference using LMC-R. (A) Kernel density estimates of parameter posteriors; 6000 iterations were excluded as burn-ins, out of 20,000 samples. The red line indicates the true parameter.

**Fig. 5:**
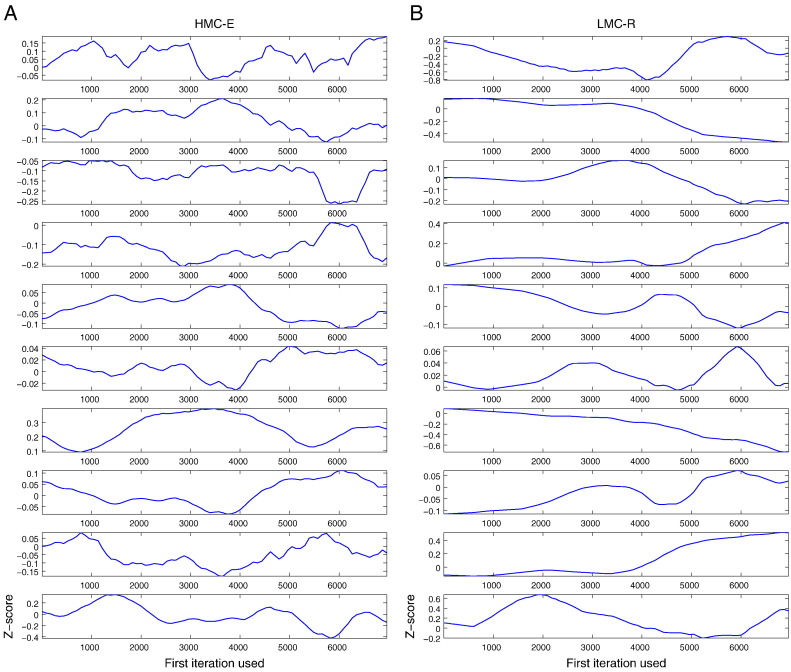
Geweke’s convergence plot. The occurrence of *Z*-scores for the 10 parameters (arranged as ten rows) well within 2 standard deviations of zero does not indicate lack of convergence for (A) HMC-E and (B) LMC-R.

This inherent problem in the LMC-E can be remedied by augmenting a mass-matrix with the local geometric information such that different parameters can make variable steps during the sampling procedure. A simpler approach is to enable Langevin diffusion of the samples using their natural gradients (see Methods). This is exactly what LMC-R does: it augments the stochastic differential equation governing the trajectory with its natural gradient, assuming that the natural gradient is locally constant. For our NMM, this amounts to a 38-fold reduction in computational time (Table 3) but with a concomitant reduction in the number of independent samples (Fig. 4). Notice that the posterior support for parameters 4 and 7 do not include the true parameter. Thus, LMC-R suffers from lower mean ESS (Table 3), while being computationally more efficient (under fixed samples) than HMC-E. Geweke’s convergence test show that for both HMC-E and LMC-R, the *Z*-scores for all 10 parameters are well within 2 standard deviations of zero (Fig. 5); this does not indicate lack of convergence.

**Fig. 6:**
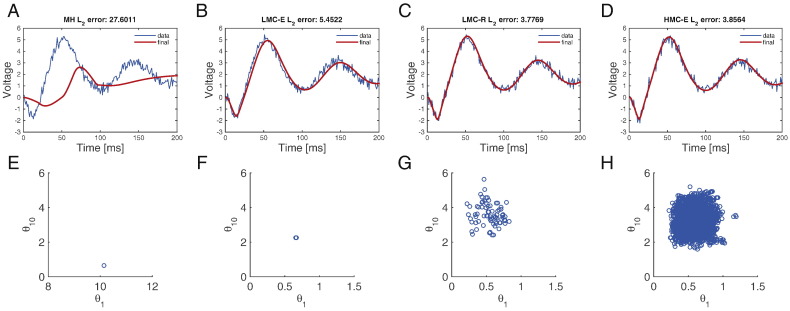
Efficiency of the MCMC methods. (A) Predicted voltage using the posterior mean computed from 14,000 samples based on random walk Metropolis–Hastings algorithm. (B) Same as A but with the LMC-E algorithm. (C) Same as A but with the LMC-R algorithm. (D) Same as A but with the HMC-E algorithm. (E) Schematic displaying total samples drawn from the posterior density using the MH algorithm. Parameters 1 and 10 are plotted. (F) Same as E but using the LMC-E algorithm. (G) Same as E but using the LMC-R algorithm. (H) Same as E but using the HMC-E algorithm.

**Fig. 7:**
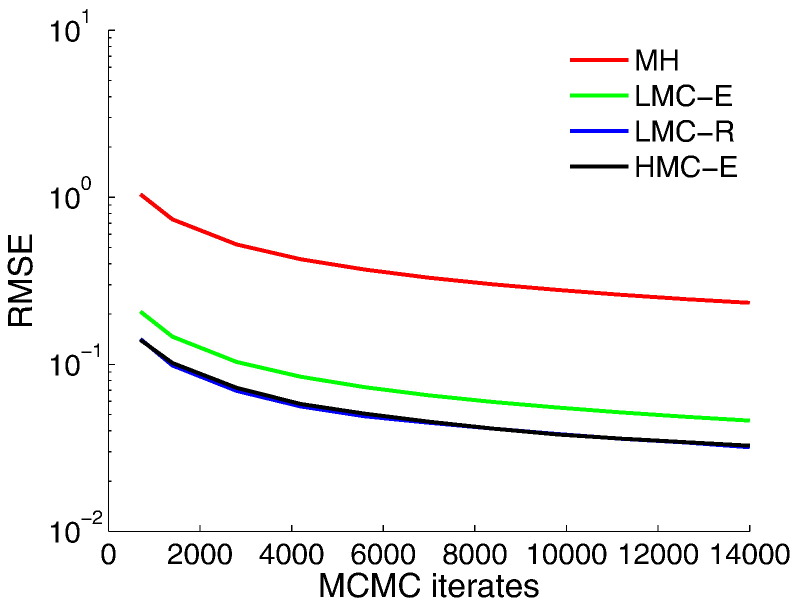
Root mean-squared error (RMSE) of the posterior prediction. RMSE of posterior prediction over 20,000 samples, where 6000 samples were discarded as burn-in iterations.

Comparison of the *l*_2_ error norm (Fig. 6A–D) shows that HMC-E and LMC-R have a similar accuracy (also see Fig. 7), which exceeds the random walk Metropolis–Hastings (MH) scheme. Taking just under 10 min to generate 20,000 samples, MH has the worst mean ESS (Table 3). This is demonstrated in Fig. 6D, where we observe that MH draws a low number of independent samples. This behaviour is dependent on the starting position of the sampler, where re-starting near the invariant distribution can reduce the *l*_2_ error norm considerably, but keeps the mean ESS unchanged. We conclude that for the problem at hand, i.e., inference in the presence of stable as well as unstable dynamics, random walk MH simply does not converge.

LMC-R, on the other hand, mixes more efficiently than MH but worse than HMC-E (Fig. 6E–H). Fig. 7 reiterates the fact that the root-mean-squared-error (RMSE) reduces as a function of number of iterations, with LMC-R and HMC-E demonstrating comparable error norms. In summary, our comparison shows that HMC-E is the most statistically efficient estimator marked by highest ESS while LMC-R is the second best. From a *l*_2_ minimization perspective, it might be more useful to use a LMC-R given that it displays similar *l*_2_ error decrease and is computationally more efficient (under fixed number of samples) in comparison to a HMC-E sampler.

**Fig. 8:**
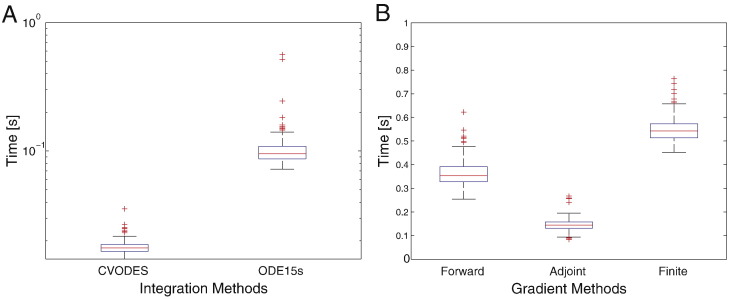
Components of an efficient MCMC scheme. (A) Average time taken to integrate a single node DCM using CVODES and ode15s. Data from 200 evaluations with the parameters sampled from the prior. Identical tolerances were used for both methods. (B) Time taken for gradient estimation using forward sensitivities, adjoint method and finite-differences. Data from 200 1-node DCMs using CVODES.

The absolute computational efficiency that we achieve in both HMC and LMC is primarily due to the use of first order gradients. This is facilitated by efficient ODE integrators (Fig. 8A), providing almost 10-fold improvement over the stiff differential equation integrator (ode15s) in MATLAB. Similarly, gradients obtained from the adjoint system prove to be almost 4 times faster in comparison to gradients based on finite differences (Fig. 8B). We stress that gradient algorithms used in this note do not use adjoints for gradient calculation (see Stability section in Sengupta et al., 2014). Computational time for HMC and LMC could be reduced further by relaxing the tolerances used for the forward, sensitivity and the adjoint equations.

## 4 Discussion

Earlier work—using a symmetric random walk Metropolis–Hastings algorithm—suggested that sampling-based DCM inversion schemes display slow chain mixing, in addition to slow convergence in high dimensions (Chumbley et al., 2007). Using a nonlinear dynamic causal model (one node NMM) as an exemplar, we compared the sampling performance of two gradient-based MCMC samplers. We find that in comparison to a random walk Metropolis–Hastings sampler, these schemes converge to the posterior density in a statistically efficient manner. Specifically, the HMC algorithm (with an Euclidean metric) yields a worse time-normalised effective sample size (nESS), being computationally expensive even with sophisticated parameter tuning, albeit being statistically the most efficient sampler. In contrast, the differential geometry-based LMC (with a Riemannian metric) appears to be much more suitable for inversion of this DCM—at the cost of displaying nESS that is 96% lower than that of the HMC.

An important issue—when using MCMC for Bayesian inference—is determining when the chain has converged. This criterion is crucial and therefore forms a large part of ongoing research that ascertains rapid convergence. Running a MCMC sampler for a long time will result in ‘convergence to distribution’ at the cost of non-finite execution time. Measure-theoretic analysis of most MCMC samplers give an estimate of the number of samples required to ensure convergence, according to a total variation distance (with a specified tolerance bound) to the true posterior density. For empirical problems, this is seldom possible. A simpler but computationally wasteful strategy involves running multiple—yet independent—chains and ensuring that the posterior density obtained by each chain is identical in terms of its lower moments. A more cogent diagnostics to estimate convergence of the Markov chain uses the normal theory approximations of Gelman and Rubin (1992). This introduces a shrink factor that tends to 1 (depending on between-chain and within-chain convergence) as the Markov chain converges. Unfortunately, a clinical neuroimager may not have the luxury of a high-performance computer; therefore, such a method based on multiple chains may not be suitable in a clinical setting. Therefore, it might be more prudent to limit ourselves to single chain metrics such as that of Geweke (1992) (used in this study) or Raftery and Lewis (1992), even if they are univariate convergence indicators. For a discussion of convergence estimators, see Table 1 in Cowles and Carlin (1996).

The fidelity of MCMC samplers can therefore be gauged under two indicators—(a) sampling efficiency in terms of average number of independent samples generated (post-convergence) and (b) computational efficiency under the generation of a fixed number of samples. For example, MH and LMC-E have clearly not converged due to their intrinsic inability to steer the domains of dynamical instability, a fundamental character of the underlying DCM. This makes the underlying comparison of sampling efficiency (using nESS) meaningless. Our results suggest that under the specific generative model that we have adopted (with unstable dynamics), using MH or LMC-E is simply inappropriate. It is vital to appreciate that computational efficiency under a fixed number of samples is distinct from computational efficiency under variable number of samples drawn, until convergence.

Our parameter selection scheme uses Gaussian process (GP) optimisation to derive and bound the parameter acquisition function that governs how the next time-step and leapfrog steps are selected (Srinivas et al., 2010, Srinivas et al., 2012). Heuristically, optimising the tuning parameters of the HMC algorithm is difficult, with algorithms such as the no-U-turn sampler (NUTS) (Hoffman and Gelman, 2014) representing the best remedy. However, bounding the cumulative performance—in terms of its maximal information gain—appears to be an alternative approach. Beskos et al. (2013) have shown how the cost of generating a proposal in HMC changes as a function of the integrator step-size. With an acceptance probability of 65.1% HMC requires $O\left(d^{1/4}\right)$ steps to traverse the state-space as dimension *d* → ∞. To apply Bayesian optimisation, as we have done here, employ no-U-turn sampler (Hoffman and Gelman, 2014) as used in Stan (Stan Development Team, 2014) or use partial momentum refreshment (Mudigonda et al., 2014) may seem unprincipled although all of them have enjoyed some empirical success. A principled criterion can only be established by optimising the natural parameters of the symplectic integrator. In fact, some recent work in understanding the geometry behind Hamiltonian Monte Carlo point to the fact that optimising the integrator step and the number of integrator steps may lead to inefficiency in the Hamiltonian flow (Betancourt et al., under review). Betancourt et al. (under review) argue that optimising integrator step size and the number of integrator steps may lead to short integration time, limiting the efficacy of the underlying Hamiltonian flow. Betancourt et al. (2015) also argue that the step size motivated by ad hoc optimisation strategies can be much larger than those guaranteeing topological stability and vanishing asymptotic error. The practitioner should therefore be wary of these facts, employing domain-specific experience to cross-validate against multiple optimisation criteria.

To attain lower nESS, an extensive amount of work has culminated in the use of second-order geometric information in the form of Christoffel symbols, i.e., the derivatives of the metric tensor, in manifold MALA (MMALA) (Byrne and Girolami, 2013, Girolami and Calderhead, 2011, Lan et al., 2014). This relaxes the locally constant metric assumption that we have in place in this note. Significant progress has also been made in using information geometry based augmentation of the HMC algorithm—in the form of the Riemannian metric manifold HMC (HMC-R) algorithm (Byrne and Girolami, 2013, Girolami and Calderhead, 2011, Lan et al., 2014). This brings us to the vital issue of scalability of these inference algorithms—do the MCMC methods tested in this paper scale for DCMs with tens of nodes? With the implementation presented in this paper, this seems difficult as calculating the gradient of the metric tensor itself amounts to tens of minutes of compute time, on a conventional workstation. Of course, using a high-performance cluster reduces this time; we operate under the assumption that only few statistical parametric mapping (SPM) users do have access to such clusters. From an optimisation point of view, the drift term of the LMC algorithm can be interpreted as a scaled Newton step (Nocedal and Wright, 2006), specifically a Gauss–Newton approximation of the Hessian. To improve sampling efficiency, MMALA in general uses a non-constant metric tensor, constructing such operators requires third-order derivatives of negative log posterior density. If one were to take the second-order terms into consideration, the algorithm becomes comparable to the full Newton method, i.e., in a model with *P* parameters, a Hessian matrix stored in single precision would approximately require 4*P*^2^ bytes of memory. Just like in the standard Newton method, constructing the metric tensor and the associated derivatives prove to be expensive. As we show in our forthcoming paper (Sengupta et al., in preparation), this calculation can be made computationally efficient by using two constructs—(a) adjoined formulation of the gradients and the Hessians (Sengupta et al., 2014) and (b) Karhunen–Loève expansion of the Fisher-information matrix. This efficiency is simply due to the fact that the Fisher information matrix can be calculated as a solution to an adjoint equation.

The computational efficiency of gradient-based samplers comes from the added information from the gradients (Amari and Douglas, 1998, Girolami and Calderhead, 2011), efficient time integration using CVODES (Hindmarsh and Serban, 2002) and adjoined dynamic systems (Sengupta et al., 2014) that reduce the computational cost of gradient calculations (for systems that are known to be stable a priori). The exponential divergence of the states diagnosed from low joint log-likelihood protects us from stability issues, without explicit stability analysis at each MCMC step. The stability constraints that we have incorporated to make our inference problem harder just increases computing time—where the ODE integrator invariably suffers from a high condition number for the propagator, slowing down the integration process. Problems that are a priori known to be stable will yield far less compute time than reported here. Furthermore, adjoints could be used for faster gradient computations.

The workhorse of DCM inversion is the variational Laplace (VL) scheme (Friston et al., 2003, Friston et al., 2007). Our long-term goal is to derive sampling methods that provide us with posterior densities at comparable computational expenditure. This may have deep consequences for neuroscience: minimisation of variational free energy underlies both (variational and sampling) schemes (but using a different parameterisation of the approximate posterior) (Fiser et al., 2010). If the brain is also performing some form of Bayesian inference, neuronal populations either implement sampling—and we model this in terms of sufficient statistics of the population density—or neuronal populations encode the sufficient statistics per se. It may well be that certain parts of the nervous system choose to operate under a sampling formalism (MCMC) (Hennequin et al., 2014), while others adopt a different approximate formalism (variational Bayes or predictive coding). It is only with adequate experiments that such questions can be resolved; however, we first have to understand the computational anatomy of sampling per se, which is the focus of the current work.

## Figures and Tables

**Table 1:** Model parameters used for dynamic causal modelling.

Parameter	Shape (*k*_1_)	Scale (*k*_2_)	True parameters
*g*_1_	18.16	0.03	0.42
*g*_2_	29.9	0.02	0.76
*g*_3_	29.14	0.005	0.15
*g*_4_	30.77	0.007	0.16
*δ*	22.87	0.51	12.13
*τ_i_*	34.67	0.23	7.77
*h_i_*	20.44	0.96	27.88
*τ_e_*	33.02	0.16	5.77
*h_e_*	24.17	0.07	1.63
*u*	23.62	0.13	3.94

Parameters describing the prior (Gamma distribution). Also shown are the parameters for generating the raw data (Fig. 1).

**Table 2:** Simulation parameters.

Parameter	Value
Samples collected, *v*	20,000
Burn-in samples, *ϕ*	6000
Bounds on leapfrog steps, *L*	[10, 150]
Bounds on step-size, *ε*	[0.0001, 0.01]
*λ*	0.2
*K*	0.1
*ς*	2

Parameters describing the HMC sampler.

**Table 3:** Effective sample size (ESS) obtained from various samplers.

Sampler	Time (minutes)	Mean ESS (samples)	nESS (minutes/sample)	*l*_2_ error
Metropolis	9.84	1	9.84	27.6
LMC-R	70.68	10.88	15.39	3.78
LMC-E	63.48	4.04	20.31	5.45
HMC-E	2755.87	95.13	495.65	3.86

Wall-time and average ESS for 10 parameters. Worst-case time normalised ESS (nESS) is computed using the minimum ESS for each method. The prior distribution for the parameters as well as the parameters used to generate the exemplar raw data are given in Table 1. Due to the intrinsic inability of MH and LMC-E to steer away from dynamic instability (resulting in non-convergence), comparison of their respective ESS is meaningless.

## Appendix A Basic MCMC terminology

### A.1 Invariant distribution

A probability distribution *π* is called invariant if and only if πT=T, i.e., *π* is a left eigenvector for T with eigenvalue 1. T is the transition kernel (a conditional probability).

### A.2 Criteria for an invariant distribution

For the distribution of *θ_t_* to converge to its invariant or stationary distribution, the Markov chain has to be (a) *irreducible*—starting from any point, the chain can reach any non-empty set with positive probability (also known as probabilistic connectedness condition), (b) *aperiodic*—returns to a state at irregular times; this stops the chain from oscillating and (c) *positive recurrent*—if the initial *θ*_0_ is sampled from *π*(⋅), then all subsequent iterates will also be distributed according to *π*(⋅).

### A.3 Ergodicity of the Markov chain

A state is ergodic if it is aperiodic and positive recurrent, which means that the state has a period of 1 and has finite average recurrence time. If all states of a (irreducible) Markov chain are ergodic, then the chain is said to be ergodic. Consequently, a Markov chain will have a unique invariant probability density (in our case, the approximate posterior density) if and only if the states are positive Harris recurrent.

### A.4 Geometric ergodicity

The distribution of *θ* is geometrically ergodic in total variation norm if it is (a) ergodic and (b) there exists a *κ* in [0, 1) and a function V>1 s.t. $\sum_{j}\left|T_{ij}\left(t\right)-\pi \left(j\right)\right|⩽V\left(i\right)\kappa^{t}$. The smallest *κ* for which the function V exists is called the rate of convergence.

### A.5 Uniform ergodicity

An ergodic Markov chain is uniformly ergodic if there exists a function V and a finite constant *κ* in [0, 1) s.t. $\sum_{j}\left|T_{ij}\left(t\right)-\pi \left(j\right)\right|⩽V\kappa^{t}$. This is known as Doeblin’s condition or *ϕ*-mixing.

### A.6 Convergence of MH

A symmetric random walk Metropolis–Hastings (MH) algorithm cannot be uniformly ergodic when the state space is not bounded (see Theorem 3.1 and 3.2 in Mengersen and Tweedie, 1996), although it can be geometrically ergodic. Geometric ergodicity is equivalent to the acceptance probability being uniformly bounded away from zero.

### A.7 Burn-in

Burn-in refers to the practice of discarding initial iterations of a Markov chain to specify initial distributions of the form $\pi T^{\phi }$. *ϕ* is the number of burn-in iterations. Note that the strong law of large numbers and the central limit theorem holds regardless of the starting distribution.

## Appendix B Proof for Eq. (17)

Here, we present a short derivation of the manifold version of a Langevin diffusion (Eq. (17)). Let us consider a random variable *θ* with probability density *π*(*θ*) where $J\left(\theta \right)\text{≡}\log\pi \left(\theta \right)$. The time-evolution of such a variable is governed by a stochastic differential equation (SDE),

(23) $$d\theta \left(t\right)=\frac{1}{2}\nabla_{\theta }J\left(\theta \left(t\right)\right)dt+db\left(t\right)$$

Here, **b** denotes the Brownian motion. Although in theory such a SDE should converge to a unique and invariant stationary density, discretisation using numerical methods often lead to non-convergence or convergence to a ‘wrong’ stationary density. This can be alleviated by imposing a Metropolis–Hastings correction to the errors introduced by the numerical algorithms, leading consequently to the eponymous Metropolis-adjusted Langevin algorithm (MALA or LMC-E).

By virtue of the Nash embedding theorem (Nash, 1956), our aim here is to define the above mentioned (Langevin) diffusion process on a manifold M that is embedded on a higher dimensional Euclidean space ℝ^*n*^. Before we start our derivation, let us generalise the Laplace operator to Riemannian and pseudo-Riemannian manifolds. This linear operator is known as the Laplace–Beltrami operator, composed as the divergence of the covariant derivative. Assume that M is an oriented Riemannian manifold, then the volume form on M indexed by the coordinate system *θ^i^* is

(24) $${\mathrm{vol}}_{n}=\sqrt{\left|G\right|}d\theta^{1}\wedge d\theta^{2}\ldots d\theta^{n}$$

where *dθ*^*i*^ are the 1-forms forming a dual basis, *G* is the metric tensor and ∧ is the familiar wedge product. The divergence divΘ of a vector field Θ on a manifold Θ is then a scalar function with

(25) $$\left(\mathrm{div\Theta}\right){\mathrm{vol}}_{n}=L_{\Theta }{\mathrm{vol}}_{n}$$

with *L* denoting the Lie derivative of the vector field Θ. Using Einstein notation, such divergence can be written in local co-ordinates,

(26) $$\mathrm{div\Theta}=\frac{1}{\sqrt{\left|G\right|}}\partial_{i}\left(\sqrt{\left|G\right|}d\Theta^{i}\right)$$

Now for any scalar function J we can also define a vector field ∇J on the manifold using inner products for all vectors *v*_*θ*_ at point *θ* on the tangent space *T*_*θ*_M,

(27) $$\begin{array}{c} \langle \nabla J\left(\theta \right),v_{\theta }\rangle =dJ\left(\theta \right)\left(v_{\theta }\right)\\ {\left(\tilde{\nabla }J\right)}^{i}=\partial^{i}J=G^{ij}\partial_{j}J \end{array}$$

dJ is the exterior derivative. With these identities, the Laplace–Beltrami operator (∆) reads,

(28) $$\Delta J=\frac{1}{\sqrt{\left|G\right|}}\partial_{i}\left(\sqrt{\left|G\right|}G^{ij}\partial_{j}J\right)$$

The Langevin Eq. (23) consists of two terms—a drift term and a diffusion term where the latter is represented by the Laplace–Beltrami operator (diffusion with ‘constant’ infinitesimal variance with respect to the metric) when diffusion occurs on a Riemannian manifold. While the gradient obtains the form determined by Eq. (27), using Ito’s calculus one can show the nonlinear mapping of the martingale *d***b**(*t*) becomes,

(29) $$d{\tilde{b}}_{t}=\frac{1}{2}\frac{1}{\sqrt{\left|G\right|}}\partial_{i}\left(\sqrt{\left|G\right|}G^{ij}\right)dt+\sqrt{G^{ij}}db_{t}$$

The probability density *π*(*θ*) also has to be corrected to yield the correct invariant density; this can be attained by the following transformation

(30) $$\tilde{\pi }\left(\theta \right)=\pi \left(\theta \right)\frac{1}{\sqrt{\left|G\left(\theta \right)\right|}}$$

Combining all the transformations, we obtain Eq. (17). Also refer to Xifara (2014) and Livingstone (2014) for more details (private communication; Sam Livingstone).

## Appendix C Proof for Eq. (19)

Fisher information comes in a variety of forms—for example, the *observed Fisher information* is simply the negative Hessian of the log target distribution; regrettably, such a quantity does not preserve the inner product on the manifold, especially so when the score vector is not zero. Such a metric cannot also be guaranteed to be positive definite, diluting the meaning of a metric tensor. Another approximation, the *empirical Fisher information* is proven to be positive definite although its convergence could be challenged in the small sample size limit. The *expected Fisher information*, on the other hand, turns out to be invariant to reparameterization of the data while its inverse gives us the attainable asymptotic performance of an unbiased estimator. Therefore, the metric *G* in Appendix B is taken as an approximation of the true Fisher information metric. Specifically, it is the expected Fisher information in combination with the negative hessian of the log-prior. With *π*_*post*_(*θ*|*y*) ∝ *π*_*like*_(*y*|*θ*)*π*_*prior*_(*θ*) the metric reads,

(31) $$G\left(\theta \right)=E_{y\left|\theta \right)}\left[-\nabla^{2}\log\pi_{\mathrm{like}}\left(y\left|\theta \right)\right)\right]-\nabla^{2}\log\pi_{\mathrm{prior}}\left(\theta \right)$$

Expanding this equation and keeping the first order terms leads to Eq. (19).
